# Hesperidin Prevents Nitric Oxide Deficiency-Induced Cardiovascular Remodeling in Rats via Suppressing TGF-β1 and MMPs Protein Expression

**DOI:** 10.3390/nu10101549

**Published:** 2018-10-19

**Authors:** Putcharawipa Maneesai, Sarawoot Bunbupha, Prapassorn Potue, Thewarid Berkban, Upa Kukongviriyapan, Veerapol Kukongviriyapan, Parichat Prachaney, Poungrat Pakdeechote

**Affiliations:** 1 Department of Physiology, Faculty of Medicine, Khon Kaen University, Khon Kaen 40002, Thailand; putcma@kku.ac.th (P.M.); pairpassorn@gmail.com (P.P.); upa_ku@kku.ac.th (U.K.); 2 Cardiovascular Research Group, Khon Kaen University, Khon Kaen 40002, Thailand; parpra@kku.ac.th; 3 Faculty of Medicine, Mahasarakham University, Maha Sarakham 44000, Thailand; bugvo@hotmail.com (S.B.); no_ng_pt@hotmail.com (T.B.); 4 Department of Pharmacology, Faculty of Medicine, Khon Kaen University, Khon Kaen 40002, Thailand; veerapol@kku.ac.th; 5 Department of Anatomy, Faculty of Medicine, Khon Kaen University, Khon Kaen 40002, Thailand

**Keywords:** hesperidin, l-NAME, cardiovascular remodeling, oxidative stress, inflammation

## Abstract

Hesperidin is a major flavonoid isolated from citrus fruits that exhibits several biological activities. This study aims to evaluate the effect of hesperidin on cardiovascular remodeling induced by n-nitro l-arginine methyl ester (l-NAME) in rats. Male Sprague-Dawley rats were treated with l-NAME (40 mg/kg), l-NAME plus hesperidin (15 mg/kg), hesperidin (30 mg/kg), or captopril (2.5 mg/kg) for five weeks (*n* = 8/group). Hesperidin or captopril significantly prevented the development of hypertension in l-NAME rats. l-NAME-induced cardiac remodeling, i.e., increases in wall thickness, cross-sectional area (CSA), and fibrosis in the left ventricular and vascular remodeling, i.e., increases in wall thickness, CSA, vascular smooth muscle cells, and collagen deposition in the aorta were attenuated by hesperidin or captopril. These were associated with reduced oxidative stress markers, tumor necrosis factor-alpha (TNF-α), transforming growth factor-beta 1 (TGF-β1), and enhancing plasma nitric oxide metabolite (NOx) in l-NAME treated groups. Furthermore, up-regulation of tumor necrosis factor receptor type 1 (TNF-R1) and TGF- β1 protein expression and the overexpression of matrix metalloproteinase-2 (MMP-2) and matrix metalloproteinase-9 (MMP-9) was suppressed in l-NAME rats treated with hesperidin or captopril. These data suggested that hesperidin had cardioprotective effects in l-NAME hypertensive rats. The possible mechanism may involve antioxidant and anti-inflammatory effects.

## 1. Introduction

Nitric oxide (NO) is a crucial vasodilator derived from vascular endothelium to regulate vascular tone [1]. A reduction of NO production results in increased vascular resistance and high blood pressure. n^ω^-nitro l-arginine methyl ester (l-NAME), an l-arginine analogue, is widely used as an inhibitor of nitric oxide synthase (NOS) activity to represent an animal model of hypertension. It has been reported that l-NAME-induced hypertension in rats is characterized by insufficient NO production, increased systemic oxidative stress, inflammation, and endothelial dysfunction [2]. Furthermore, l-NAME-induced hypertension and cardiovascular remodeling have also been reported in rats. For example, the administration of l-NAME (40 mg/kg) for four or five weeks causes high blood pressure and cardiovascular remodeling, including left ventricular hypertrophy, myocardial fibrosis, and thickening of the vascular wall [3,4,5]. It is generally known that the main sequel of cardiovascular remodeling is heart failure, which is the major cause of death worldwide [6].

The initial stage of cardiac remodeling is myocardial hypertrophy because of the adaptive response to a high-pressure load to preserve cardiac function and obtain normal cardiac work. In addition, the cardiac remodeling process in l-NAME-treated rats is involved in the production of myocardial fibrosis [7]. There are substantial data to show the molecular mechanism of extensive areas of cardiac fibrosis which is associated with the activation of various downstream inflammatory [8] and oxidative stress initiatives [9,10]. For example, a high level of tumor necrosis factor (TNF-α), a pro-inflammatory cytokine, developed in response to oxidative stress in l-NAME-induced hypertension has been reported [4,11]. These inflammatory responses subsequently activate the profibrotic mediator of the transforming growth factor β1 (TGF-β1) [11]. It is well-established that TGF-β1 plays a key role in fibrogenesis by activating apoptosis, collagen, and matrix protein synthesis [12,13,14]. For vascular structural changes in hypertension, it is known to be an adaptive response to an increase in wall tension [15]. This response is also related to the extracellular matrix degradation of elastic fibers since the up-regulation of matrix metalloproteinase-2 (MMP-2) and matrix metalloproteinase-9 (MMP-9) expression in vessel tissue has been confirmed in animal models of hypertension. Several lines of evidence have indicated that the activation of MMP-2/9 protein expression found in the vascular remodeling process is mediated by the inflammatory cytokine, TNF-α [16,17,18]. Thus, it is noteworthy that natural products with high antioxidant and anti-inflammatory activities might be useful for alleviating cardiovascular alterations induced by nitric oxide deficiency.

Captopril is an angiotensin-converting enzyme (ACE) inhibitor and is commonly used as an anti-hypertensive drug [19]. Its mechanism of action has been well-documented to reduce angiotensin II production, which subsequently suppresses the renin-angiotensin-aldosterone system (RAAS) [19]. Other possible anti-hypertensive mechanisms include increased bradykinin and prostaglandins levels [20], the inhibition of superoxide production [21], and the free radical scavenging effect [22]. Many studies have already reported on the cardiovascular effects of captopril in nitric oxide-deficient hypertensive rats, i.e., lowering high blood pressure, improving vascular function [21], and preventing cardiovascular remodeling [23]. In l-NAME hypertensive rats, there is evidence showing the up-regulation of angiotensin II receptor type 1 (AT1R) which mediates nicotinamide adenine dinucleotide phosphate (NADPH) oxidase expression and superoxide formation [10]. This study used captopril as a positive control agent because the l-NAME hypertension model is also involved in the activation of the RAAS, where captopril inhibits the RAAS.

Hesperidin is a flavanone glycoside, a subclass of flavonoids, abundantly found in citrus fruits such as lemon or orange peels or juices [24]. Numerous beneficial effects of hesperidin have been published. For example, the antioxidant effect of hesperidin has been reported to be able to sequester 1,1-diphenyl-2-picrylhydrazyl (DPPH) and protect cell injury-induced by paraquat and hydrogen peroxide [25], reduce plasma levels of lipid peroxidation markers, and increase antioxidant enzyme activities in heart tissue in experimentally ischemic myocardial rats [26]. Hesperidin has also exhibited an anti-inflammatory effect by reducing circulating inflammatory markers, i.e., TNF-α, interleukin 6 (IL-6), and a high-sensitivity C-reactive protein (hs-CRP), in patients with type 2 diabetes [27] and suppressed inflammatory responses in lipopolysaccharide-induced RAW 264.7 cells [28]. Subsequently, a clinical study revealed that a combination of hesperidin, diosmin, and troxerutin was effective in relieving the symptoms of acute hemorrhoidal disease [29]. Recently, the current authors have demonstrated an anti-hypertensive effect of hesperidin in renovascular hypertensive rats that involved the suppression of the renin-angiotensin system [30]. This study was intended to further explore whether hesperidin could prevent l-NAME-induced hypertension and cardiovascular remodeling in rats.

## 2. Materials and Methods

### 2.1. Drugs and Chemicals

Hesperidin (purity ≥ 98%) was purchased from Chem Faces Company (Wuhan, Hubei, China). n(g)-Nitro-l-arginine methyl ester hydrochloride (l-NAME) and captopril were purchased from Sigma-Aldrich Corp (St. Louis, MO, USA). All the other chemicals used in this study were obtained from standard companies and were of analytical grade quality.

### 2.2. Animals and Experimental Protocols

Male Sprague-Dawley rats (body weight 220–250 g) were supplied by Nomura Siam International Co., Ltd., Bangkok, Thailand. The animals were housed in a Heating, Ventilation and Air-Conditioning (HVAC) System (25 ± 2 °C) facility and maintained on a 12 h light and 12 h dark cycle with free access to a standard rat diet and water at the Northeast Laboratory Animal Center, Khon Kaen University. All the experimental protocols in this study were in accordance with the standards for the care and use of experimental animals and approval for all the experiments was obtained from the Animal Ethics Committee of Khon Kaen University, Khon Kaen, Thailand (AEKKU-NELAC 37/2559).

After a seven-day acclimatization period, the rats were randomly assigned to 5 groups (8/group). The control group animals received tap water and were orally administrated propylene glycol (PG, 1.5 mL/Kg) as a vehicle. l-NAME treated rats received l-NAME (40 mg/kg/day) in their drinking water and were further divided into the following 4 groups; l-NAME plus PG, l-NAME plus hesperidin at a dose of 15 mg/kg (l-NAME + H15 group), l-NAME plus hesperidin 30 mg/kg (l-NMAE + H30 group), l-NAME group plus captopril at a dose of 2.5 mg/kg (l-NAME + Cap group). Additionally, normal rats (*n* = 5) were orally treated with hesperidin (30 mg/kg) for 5 weeks to test the hypotensive effect of hesperidin. Hesperidin and captopril were dissolved in vehicle and intragastrically administered once daily for five weeks. The doses of hesperidin and captopril used in this study were influenced by previous studies in this laboratory [10,30].

### 2.3. Blood Pressure Measurements

To monitor blood pressure changes throughout the experimental period, systolic blood pressure (SP) was obtained in awake rats once a week for 5 weeks using tail-cuff plethysmography (IITC/Life Science Instrument model 229 and model 179 amplifier; Woodland Hills, CA, USA). At the end of the final experimental day, the rats were anesthetized with pentobarbital sodium (60 mg/kg, ip.). Then, the femoral artery was cannulated and connected to a pressure transducer for monitoring the baseline values of SP, diastolic blood pressure (DP), mean arterial pressure (MAP), and heart rate (HR) using the Acknowledge Data Acquisition software (Biopac Systems Inc., Santa Barbara, CA, USA).

### 2.4. Collection of Blood and Organs

After the blood pressure measurement, the rats were sacrificed by exsanguination and blood samples were collected from abdominal aortas into Ethylenediaminetetraacetic acid (EDTA) or heparin tubes for assays of oxidative stress and inflammatory markers. The carotid arteries were rapidly excised for analysis of superoxide (O_2_^•−^) production. The thoracic aortas and heart tissues were collected for western blotting and morphometric analysis.

### 2.5. Assays of Vascular O_2_^•−^ Production, Plasma Malondialdehyde (MDA), Plasma Nitric Oxide Metabolite (Nitrate/Nitrite, NOx), Plasma TNF-α and Plasma TGF- β1 Levels 

The carotid arteries were cleaned of connective tissues, cut into 0.5 cm lengths, and incubated with 1 mL oxygenated Krebs-KCl solution at pH 7.4, 37 °C for 30 min. The production of O_2_^•−^ in the carotid arteries was determined by lucigenin-enhanced chemiluminescence, as previously described [31], with some modifications [32]. Plasma NOx was assayed using an enzymatic conversion method [33], with some modifications [32]. The concentrations of plasma TNF-α and TGF-β1 were measured using enzyme-immunoassay assay (ELISA) kits (eBioscienc, Inc., San Diego, CA, USA and ab119557, Abcam Plc, Cambridge, UK).

### 2.6. Morphometric Analysis of Thoracic Aorta and Heart Tissue

Heart weight (HW) and left ventricular weight (LVW) were measured, and calculated as an LVW/BW ratio. Thereafter, the left ventricles and thoracic aortas were fixed with 4% paraformaldehyde and then embedded in paraffin and cut into serial 5-μm-thick sections. Each section was stained with hematoxylin and eosin (H&E) and/or Picrosirius Red. Sections were captured with a Digital sight DS-2MV light microscope (Nikon, Tokyo, Japan) or a stereoscope (Nikon SMZ745T with NIS-elements D 3.2, Tokyo, Japan). Morphometric evaluations of the sections were performed with Image J software (National Institutes of Health, Bethesda, MD, USA).

### 2.7. Western Blot Analysis of Tumor Necrosis Factor Receptor 1 (TNF-R1), TGF- β1, MMP-2 and MMP-9 Protein Expressions in Cardiac and Aortic Tissues 

Protein samples were prepared through the homogenization of cardiac and aortic tissues in a lysis buffer (Cell Signaling Technology Inc., Danvers, MA, USA). The proteins were then electrophoresed on a sodium dodecylsulfate polyacrylamide gel electrophoresis system and transferred to a polyvinylidene fluoride membrane (Millipore Corporation, Bedford, MA, USA). The membranes were blocked with 5% skimmed milk in Tris-buffered saline (TBS) with 0.1% Tween 20 for 2 h at room temperature before overnight incubation at 4 °C with primary antibodies against TNF-R1, TGF-β1, MMP-2, MMP-9, or β-actin (Santa Cruz Biotechnology, Inc., Santa Cruz, CA, USA). Thereafter, the membranes were washed three times with TBS and then incubated for 2 h at room temperature with a horseradish peroxidase conjugated secondary antibody. The protein bands were detected using Luminata™ Forte horseradish peroxidase (HRP) detection reagent (Merck KGaA, Darmstadt, Germany) and the densitometric analysis was performed using ImageQuantTM 400 (GE Healthcare Life Sciences, Piscataway, NJ, USA). The intensity of each band was normalized to that of β-actin, and data were expressed as a percentage of the values determined in the control group from the same gel.

### 2.8. Statistical Analysis

Data are expressed as mean ± S.E.M. The differences among the treatment groups were analyzed through a one-way analysis of variance (ANOVA) followed by Bonferini’s post-hoc test. A *p*-value of less than 0.05 was considered as statistically significant.

## 3. Results

### 3.1. Effects of Hesperidin and Captopril on Blood Pressure in Conscious Rats

**Figure 1:**
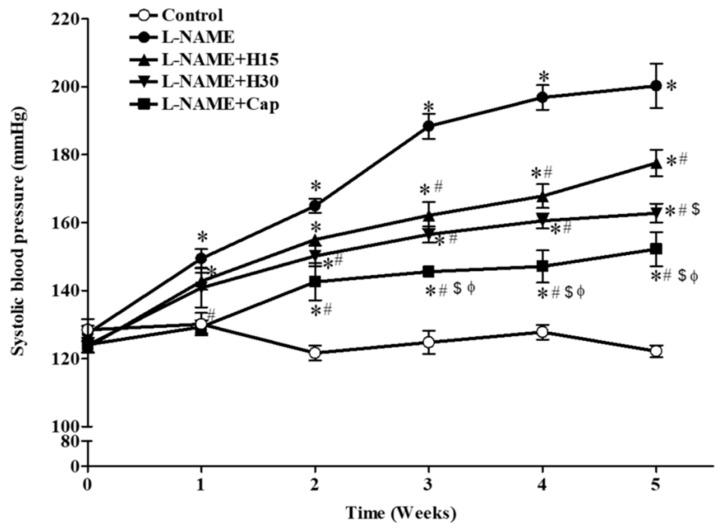
Time-course changes in systolic blood pressures of all experimental groups. Data are expressed as mean ± S.E.M (*n* = 7–8/group), * *p* < 0.05 vs. control, ^#^ *p* < 0.05 vs. l-NAME, ^$^ *p* < 0.05 vs. l-NAME + hesperidin (15 mg/kg), ^Φ^ *p* < 0.05 vs. l-NAME + hesperidin (30 mg/kg) group.

There were no significant differences in the systolic blood pressure of all the rats at the beginning of the study. The administration of l-NAME caused a gradual increase in the SP of all the rats compared to the control rats (SP at 5th week, 200.21 ± 6.52 vs. 122.14 ± 1.75 mmHg, *p* < 0.01, Figure 1). The co-administration of l-NAME and hesperidin at doses of 15 or 30 mg/kg (2.5 mg/kg) partially prevented l-NAME-induced high blood pressure in a dose-dependent manner compared to that of untreated rats (SP at 5th week, 177.50 ± 3.91 and 162.74 ± 2.82 mmHg, *p* < 0.05). Captopril also partially alleviated l-NAME-induced hypertension (152.19 ± 5.01 mmHg) compared to untreated rats (*p* < 0.05). In addition, captopril produced a greater preventive effect on SP than hesperidin (15 and 30 mg/kg).

### 3.2. Effects of Hesperidin and Captopril on SP, DP, MAP, and HR in Anesthetized Rats

The blood pressure data obtained using the indirect blood pressure measurement method were consistent with the values from the direct method since l-NAME treated rats exhibited high blood pressure, including high SP, DP, MAP, and high HR compared to those of control rats (*p* < 0.05, Table 1). Hesperidin at doses of 15 and 30 mg/kg significantly decreased SP, DP, and MAP in a dose-dependent manner compared to the untreated group (*p* < 0.05). Similarly, captopril reduced the development of hypertension induced by l-NAME compared to untreated rats (*p* < 0.05). Hesperidin at a dose 30 mg/kg, however, also affected the elevation of HR compared to untreated rats (*p* < 0.05, Table 1). Furthermore, hesperidin had no effect on blood pressure in normotensive rats (SP = 122.29 ± 4.05 mmHg, *n* =4).

### 3.3. Effects of Hesperidin and Captopril on Left Ventricular (LV) Morphometry and Fibrosis

**Figure 2:**
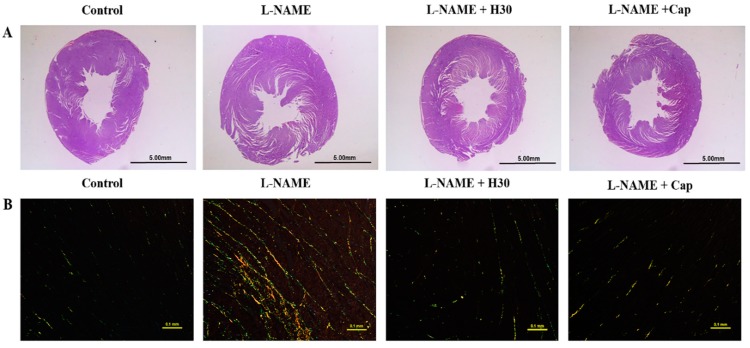
The histology and morphology of LV from control, l-NAME, l-NAME + hesperidin (30 mg/kg) and l-NAME + captopril (2.5 mg/kg) groups. Representative images of LV sections, (**A**) stained with hematoxylin and eosin under stereomicroscopes, and (**B**) stained with picrosirius red under a polarized light microscope using a 20× objective lens.

Rat body weights did not differ among all experimental groups. After 5 weeks of l-NAME administration, the HW, LVW, and LVW/BW ratios were significantly increased compared to those of control rats. The co-administration of l-NAME and hesperidin or captopril significantly decreased those values when compared to the untreated group (Table 2). Morphometric analysis of hearts showed that the chronic administration of l-NAME significantly increased LV wall thickness and LV muscle fiber cross-sectional area (CSA) compared to the normal control group (*p* < 0.05, Table 2). Hypertensive rats that received hesperidin or captopril had significantly reduced wall thicknesses and CSA of the LV compared to untreated rats (*p* < 0.05) (Table 2, Figure 2A). LV fibrosis was significantly increased in the l-NAME-treated rats compared to the normal control rats (*p* < 0.05). Hesperidin or captopril treatment significantly prevented l-NAME-induced LV fibrosis compared to the untreated rats (*p* < 0.05) (Figure 2B).

### 3.4. Effect of Hesperidin and Captopril on Vascular Morphology

**Figure 3:**
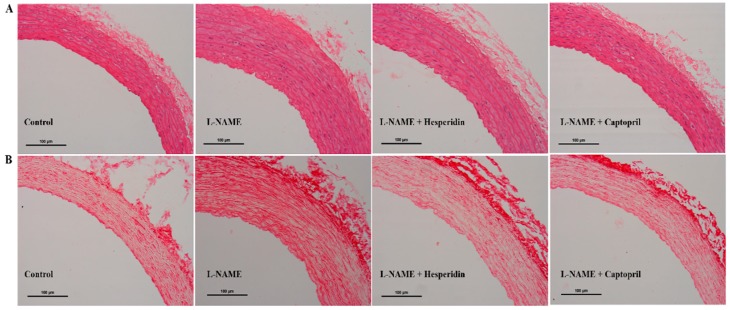
The histology and morphology of thoracic aorta from control, l-NAME, l-NAME + hesperidin (30 mg/kg), and l-NAME + captopril (2.5 mg/kg) groups. Representative images of aortic sections, (**A**) stained with hematoxylin and eosin and (**B**) stained with picrosirius red under a light microscope using a 20× objective lens.

Vascular wall hypertrophy was observed in thoracic aortas collected from l-NAME hypertensive rats (Figure 3A) with significant increases in vascular wall thickness, CSA, and smooth muscle cells numbers compared to those of the control rats (*p* < 0.05; Table 2, Figure 3A). Moreover, the relative amounts of collagen depositions (Figure 3B) in the aortic walls of l-NAME hypertensive rats were also clearly observed (*p* < 0.05; Table 2, Figure 3B). Hesperidin or captopril treatment partially prevented the vascular structural abnormalities in aortas induced by l-NAME (*p* < 0.05).

### 3.5. Effects of Hesperidin and Captopril Supplementation on Oxidative Stress Markers, Plasma Nitric Oxide Metabolites (NOx) Levels in l-NAME Treated Rats

**Figure 4:**
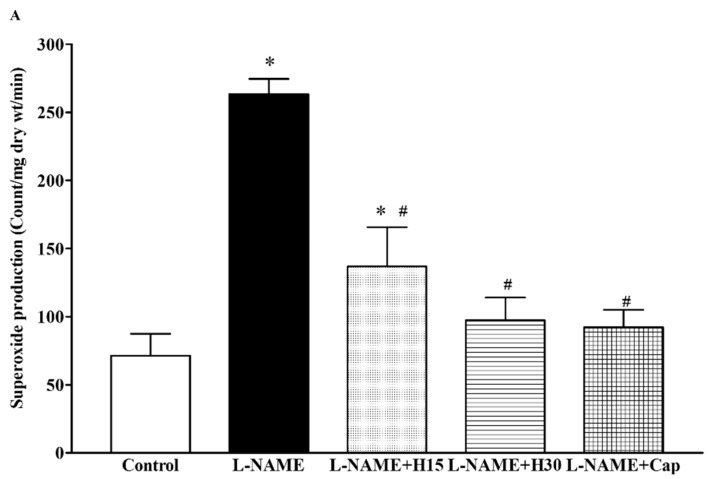

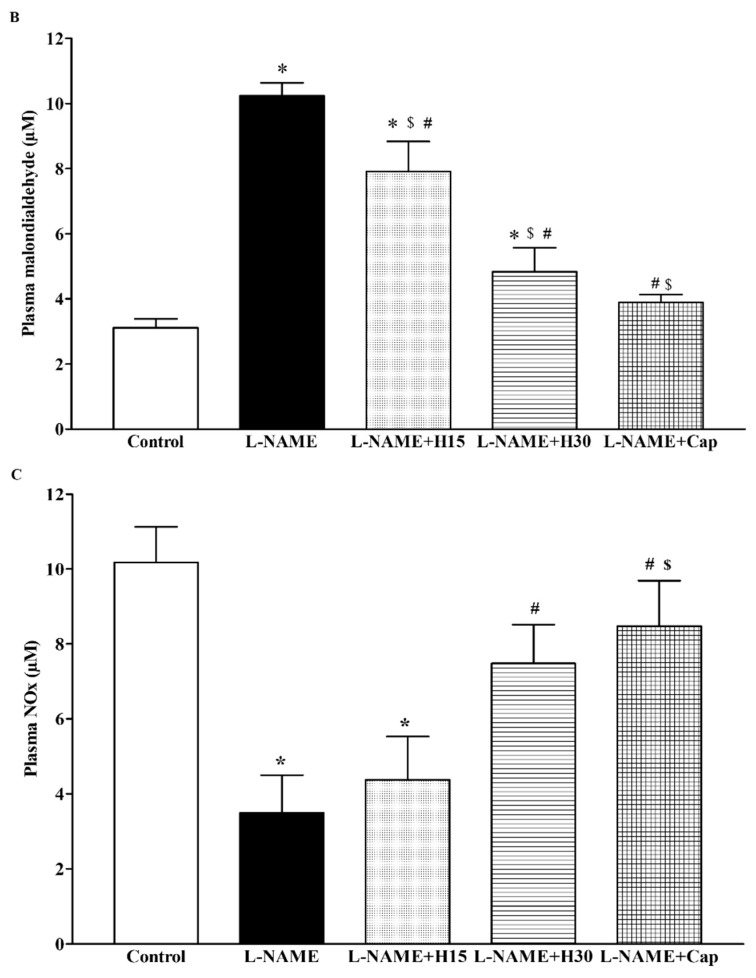
Effects of hesperidin and captopril supplementation on vascular O_2_^•−^ production, (**A**) plasma MDA (**B**) and plasma NOx (**C**) levels in control, l-NAME, l-NAME + hesperidin (15 mg/kg), l-NAME + hesperidin (30 mg/kg) and l-NAME + captopril (5 mg/kg) groups. Data are expressed as mean ± S.E.M (*n* = 7–8/group), * *p* < 0.05 vs. control, ^#^ *p* < 0.05 vs. l-NAME group, ^$^ *p* < 0.05 vs. l-NAME + H15.

l-NAME treated rats showed a significant increase in the production of vascular O_2_^•−^ (263.26 ± 11.20 vs. 71.42 ± 15.97 count/mg dry wt/min, *p* < 0.001) and plasma MDA levels compared to the control groups (10.24 ± 0.4 vs. 3.11 ± 0.27 µM, *p* < 0.05). When treated with hesperidin or captopril, the elevations of vascular O_2_^•−^ and plasma MDA were mitigated compared to those of untreated rats (7.91 ± 0.92, 4.83 ± 0.74 and 3.88 ± 0.25 count/mg dry wt/min and 138.86 ± 28.75, 97.28 ± 16.67 and 92.14 ± 12.90 µM, *p* < 0.05) (Figure 4A,B). In addition, low levels of plasma NOx were found in l-NAME hypertensive rats compared to control rats (3.49 ± 1.0 vs. 10.17 ± 0.95 µM, *p* < 0.05). These low levels of plasma NOx were improved by hesperidin or captopril supplementation (4.38 ± 1.15, 7.48 ± 1.03 and 8.48 ± 1.21 µM, *p* < 0.05) (Figure 4C).

### 3.6. Effects of Hesperidin and Captopril on Protein Expression of TNF-R1 and TGF-β1 in Heart Tissues and Concentrations of TNF-α and TGF-β1 in Plasma

**Figure 5:**
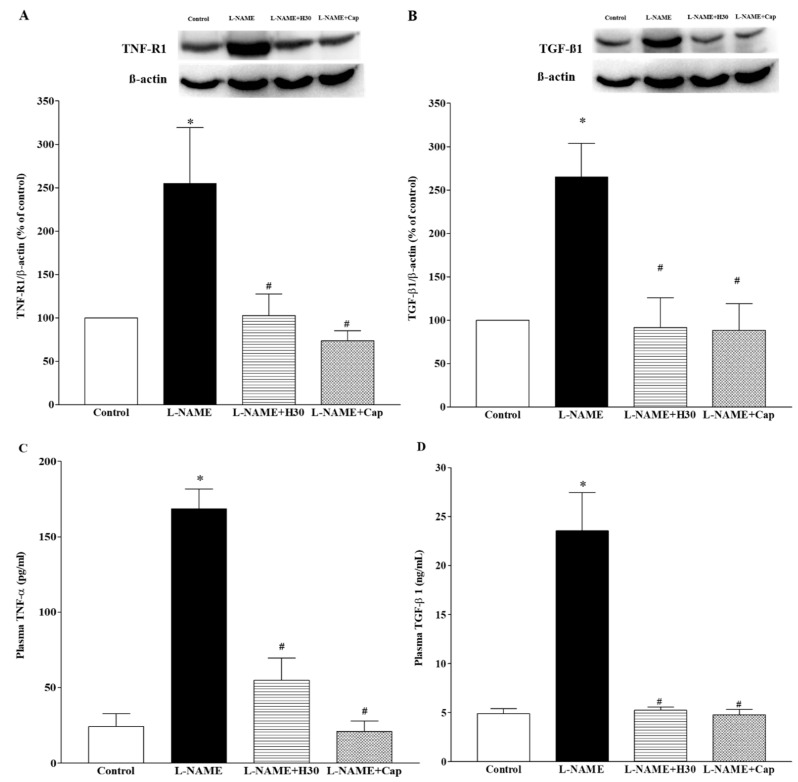
Effects of hesperidin and captopril on protein expression of TNF-R1, (**A**) and TGF-β1, (**B**) in heart tissue and on concentrations of plasma TNF-α, (**C**) and TGF-β1, (**D**) collected from control, l-NAME, l-NAME + hesperidin (30 mg/kg) and l-NAME + captopril (2.5 mg/kg) groups. The top panel shows the representative bands of TNF-R1, (**A**) and TGF-β1, (**B**) protein expression in heart tissues. Values are mean ± S.E.M (*n* = 4 for each group), * *p* < 0.05 vs. control, ^#^ *p* < 0.05 vs. l-NAME group.

Over-expressions of TNF-R1 and TGF-β1 proteins were found in heart tissues collected from the hypertensive group compared to the control group (*p* < 0.001). Interestingly, supplementation with hesperidin and captopril partially reversed these protein up-regulations (*p* < 0.01; Figure 5A,B). These results were consistent with the results in that high levels of plasma TNF-α and TGF-β1 were observed in l-NAME hypertensive rats compared to those of control rats (168.49 ± 13.05 vs. 24.21 ± 8.51 pg/mL and 23.54 ± 3.91 vs. 4.90 ± 0.50 ng/mL, *p* < 0.01). The administration of hesperidin or captopril attenuated these high levels of plasma TNF-α (58.23 ± 14.71 or 20.97 ± 6.97 pg/mL) and TGF-β1 (5.23 ± 0.32 or 4.79 ± 0.55 ng/mL, *p* < 0.05) in hypertensive rats (Figure 5C,D).

### 3.7. Effects of Hesperidin and Captopril on Protein Expression of MMP-2 and MMP-9 in Aortic Tissue

**Figure 6:**
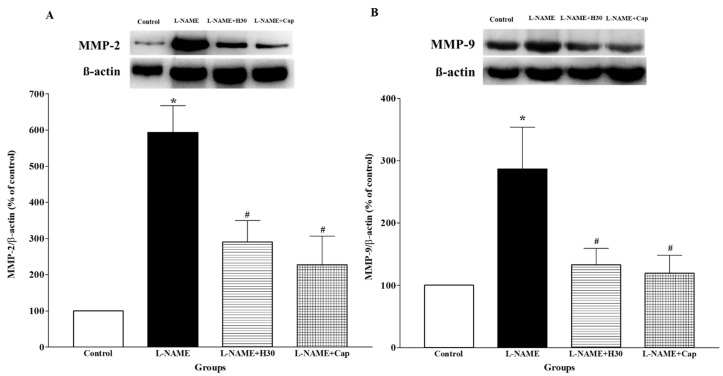
Effects of hesperidin and captopril on protein expression of MMP-2, (**A**) and MMP-9, (**B**) in aortic tissue collected from control, l-NAME, l-NAME + hesperidin (30 mg/kg) and L-NAME + captopril (2.5 mg/kg) groups. The top panels show the representative bands of MMP-2, (**A**) and MMP-9, (**B**) protein expression in thoracic aortas. Values are mean ± S.E.M (*n* = 4 for each group), * *p* < 0.05 vs. control, ^#^ *p* < 0.05 vs. l-NAME group.

A significant increase in MMP-2 and MMP-9 protein expression was observed in thoracic aortic tissues collected from the hypertensive group compared to the control group (Figure 6A,B, *p* < 0.05). Hesperidin or captopril treatment significantly suppressed the level of MMP-2 and MMP-9 protein expression compared to untreated rats, (*p* < 0.05).

## 4. Discussion

This study demonstrates that rats that received l-NAME developed hypertension and cardiovascular remodeling. Hesperidin mitigated high blood pressure and cardiac remodeling by reducing the left ventricular hypertrophy and fibrosis associated with down-regulations of TGF-β1 and TNF-R1 protein expression and a reduction of plasma TGF-β1 levels in l-NAME-induced hypertension in rats. Vascular remodeling, including vascular hypertrophy and increased collagen deposition, induced by l-NAME in rats was inhibited by hesperidin supplementation. This was consistent with the decreased protein expression of MMP-2 and MMP-9 in aortic tissue. Furthermore, hesperidin preventing cardiovascular remodeling induced by l-NAME in the present study was linked to the reduction of an inflammatory cytokine, oxidative stress markers, and enhanced NO availability.

It was found that chronic treatment of l-NAME led to the development of NO-deficient hypertension as well as cardiovascular remodeling. These remodelings included increases in LVW/HW ratio, LV wall thickness, LV CSA, LV fibrosis, aortic wall thickness, aortic cross-sectional areas, aortic smooth muscle cell numbers, and collagen deposition. It is well-accepted that the chronic inhibition of NO synthase using l-NAME results in NO depletion, increased vascular tone, and high blood pressure [34]. Several studies have demonstrated that cardiovascular remodeling occurs after chronic treatment with l-NAME (40 mg/kg) for five weeks [4,10,35]. The mechanisms involved in cardiac remodeling in an animal model of nitric oxide-deficient hypertension are still unclear; however, two possible mechanisms related to hemodynamics and non-hemodynamic aspects have been described [36]. Hemodynamic overload in hypertension provoked left ventricular hypertrophy because of the adaptive response to conserve cardiac output [37]. A reduction in NO is one of several non-hemodynamic factors that participate in cardiac remodeling because when NO is suppressed, hypertensive cardiac remodeling through the cyclic guanosine monophosphate/protein kinase G (cGMP/PKG) pathway is initiated to inhibit fibrotic synthesis [38]. It is well-documented that vascular remodeling in hypertension occurs in response to long-term modifications of hemodynamic conditions [39,40]. Furthermore, numerous studies have reported that vascular remodeling is characterized by increases in wall thickness, CSA, and smooth muscle cell numbers in l-NAME hypertensive rats [3,4,41]. In this study, hesperidin partially inhibited the development of hypertension as well as cardiovascular remodeling induced by chronic l-NAME treatment. These effects may have involved an increase in NO bioavailability, reductions of oxidative stress, and inflammation as further possibilities.

Oxidative stress is one of the important mechanisms of l-NAME-induced hypertension since l-arginine analogues activate eNOS uncoupling, leading to an overwhelming vascular superoxide generation [42]. Then, superoxide can rapidly react with nitric oxide to form peroxynitrite [43]. This reaction results in reducing nitric oxide bioavailability [44]. In the present study, increases in plasma MDA levels and vascular superoxide production were accompanied by decreased plasma NOx levels observed in the l-NAME hypertensive rats. Hesperidin alleviated l-NAME-induced oxidative stress and thus increased NO bioavailability with an increase in the plasma NOx level. Many studies have confirmed that hesperidin has a strong antioxidant activity [26,45]. Hesperidin exhibits its antioxidant properties with two main mechanisms, including directly scavenging reactive oxygen species [46], and boosting cellular antioxidant defense [25]. Thus, this is one of the possible mechanisms of the cardiovascular protective effects of hesperidin in this study that might have involved its antioxidant capability, resulting in increased NO bioavailability, which reduced vascular resistance. 

There is substantial evidence to suggest that inflammation is one of pathologies that occurs in l-NAME hypertensive rats [47,48]. The results of this study proved that, as in the previous studies, there were increases in the levels of pro-inflammatory cytokine, TNF-α, in plasma and expression of TNF-α protein in the heart tissue of l-NAME hypertensive rats. Myocardial TGF-β protein expression was also observed in l-NAME hypertensive rats. It is well-established that TGF-β plays an important role in responses to inflammation to activate fibrogenesis, which is an important pathological process for cardiac remodeling [49,50]. The present study has also shown that hesperidin attenuated cardiac remodeling, accompanied by decreased systemic and heart inflammation in l-NAME hypertensive rats. The protein expression of TGF-β in cardiac tissue was also down-regulated in the hesperidin supplemented group. The anti-inflammatory effect of hesperidin has been clearly revealed in both cellular and animal models. In human umbilical vein endothelial cells, hesperidin significantly suppressed TNF-α [51]. Li and coworkers demonstrated that hesperidin decreased the production of IL-1β, IL-6, and TNF-α in a rat model of rheumatoid arthritis [52]. Thus, the current results confirmed that the cardiprotective effect of hesperidin was associated with its great anti-inflammatory effect.

Additionally, vascular remodeling with collagen deposition was associated with the overexpression of MMP-2 and MMP-9 in aortic tissue in l-NAME hypertensive rats, as shown in this study. Several studies report that MMPs play an important role in physiological processes that contribute to hypertension-induced maladaptive arterial changes and sustained hypertension [53,54]. The overexpression of MMP-mediated vascular remodeling was stimulated by oxidative stress and inflammatory cytokines [54]. Del Mauro and coworkers demonstrated that MMP-2 and MMP-9 activity was a pathologic process in l-NAME-induced morphometric alterations in the aorta [55]. Interestingly, the authors of the present study first reported l-NAME-induced hypertension and vascular remodeling in rats in which there was an up-regulation of MMP-2 and MMP-9 protein expression in response to oxidative stress. Hesperidin prevented vascular remodeling induced by l-NAME associated with the down-regulation of MMP-2 and MMP-9. This effect might be involved in its antioxidant and anti-inflammatory effects, which further inhibited MMP activation and collagen degradation. 

Captopril was used as a positive control to prevent the development of hypertension and cardiovascular remodeling. These findings are supported by previous studies that found that captopril prevented high blood pressure, left ventricular hypertrophy, and vascular remodeling induced by l-NAME in rats [56,57]. Captopril also reduced oxidative stress and inflammatory markers and suppressed protein expressions of TNF-R1, TGF-β1, and MMPs. An antioxidative effect of captopril in the present study might be associated with two main mechanisms, direct and indirect effects. Captopril contains free sulfhydryl groups that directly scavenge oxygen free radicals [58], or it suppresses AT1R-mediated NADPH oxidase expression and superoxide production [10]. It has been demonstrated that captopril improved ventricular hypertrophy in rats by suppressing MMP-2 and MMP-9 expression [59]. In addition, an anti-inflammatory effect of captopril in the animal model of hypertension has been reported [60].

In conclusion, the findings of this study indicated that hesperidin had cardiovascular protective effects by preventing the l-NAME-induced development of hypertension and cardiovascular remodeling in rats. These effects were affirmed by reducing oxidative stress and inflammation.

## Figures and Tables

**Table 1:** Effects of hesperidin and captopril on blood pressure and heart rate in anesthetized rats.

Parameters	Control	l-NAME	l-NAME + H15	l-NAME + H30	l-NAME + Cap
SP (mmHg)	120.92 ± 2.27	205.88 ± 3.19 *	179.38 ± 16.51 *^,#^	154.07 ± 4.88 *^,#,$^	140.14 ± 7.06 ^#,$^
DP (mmHg)	72.68 ± 3.31	141.65 ± 5.73 *	114.13 ± 16.57 *^,#^	86.89 ± 5.74 *^,#,$^	91.48 ± 7.36 ^#,$^
MAP (mmHg)	88.76 ± 2.47	161.41 ± 4.01 *	135.88 ± 16.00 *^,#^	109.28 ± 5.39 *^,#,$^	107.70 ± 6.27 ^#,$^
HR (beat/min)	367.86 ± 11.90	419.30 ± 11.96 *	391.93 ± 14.35	351.44 ± 13.47 ^#,$^	384.28 ± 17.31

SP: systolic blood pressure; DP: diastolic blood pressure; MAP: mean arterial pressure; HR: heart rate. Values are mean ± S.E.M (*n* = 7–8/group), * *p* < 0.05 vs. control, ^#^ *p* < 0.05 vs. l-NAME, ^$^ *p* < 0.05 vs. l-NAME + H15.

**Table 2:** Effect of hesperidin and captopril on the cardiac mass indices and cardiovascular structural modifications in left ventricle and thoracic aorta.

**Cardiac Mass Indices**						
**Groups**	**Body Weight (g)**		**Heart Weight/BW (mg/g)**		**LVW/BW (mg/g)**	
**Control**	434 ± 6.8		3.14 ± 0.17		2.06 ± 0.10	
**l-NAME**	413 ± 16.9		4.21 ± 0.26 *		3.04 ± 0.18 *	
**l-NAME + H30**	406 ± 9.7		3.11 ± 0.23 ^#^		2.23 ± 0.17 ^#^	
**l-NAME + Cap**	401 ± 9.7		3.12 ± 0.18 ^#^		2.07 ± 0.12 ^#^	
**Left Ventricle**						
**Groups**	**LV Wall Thickness (mm)**		**LV CSA (mm^2^)**		**LV Fibrosis (%)**	
**Control**	2.72 ± 0.05		57.58 ± 1.05		0.69 ± 0.04	
**l-NAME**	3.28 ± 0.04 *		72.42 ± 0.51 *		2.72 ± 0.15 *	
**l-NAME + H30**	2.90 ± 0.06 ^#^		61.12 ± 1.75 ^#^		0.92 ± 0.09 ^#^	
**l-NAME + Cap**	2.79 ± 0.09 ^#^		59.87 ± 1.63 ^#^		1.00 ± 0.06 ^#^	
**Thoracic Aorta Structural Modifications**						
**Groups**	**Wall Thickness (µm)**	**CSA** **(×10^3^ µm^2^)**		**VSMCs** **(cells/CSA)**		**Collagen Deposition** **(% Area Fraction)**
**Control**	106.39 ± 1.02	579.00 ± 15.16		1298.00 ± 73.64		15.78 ± 0.70
**l-NAME**	150.58 ± 2.09 *	810.50 ± 18.64 *		2013.71 ± 51.62 *		31.32 ± 1.00 *
**l-NAME + H30**	127.11 ± 2.90 *^,#^	617.95 ± 18.65 ^#^		1540.16 ± 46.88 *^,#^		24.84 ± 0.69 *^,#^
**l-NAME + Cap**	129.91 ± 6.50 *^,#^	658.38 ± 40.22 ^#^		1671.78 ± 24.90 *^,#^		23.68 ± 0.63 *^,#^

LV: left ventricular, LVW: left ventricular weight, BW: body weight, CSA: cross-sectional area, VSMCs: vascular smooth muscle cells. Values are expressed as mean ± S.E.M, (*n* = 6/group). * *p* < 0.05 when compared to the control group, and**^#^***p* < 0.05 when compared to the l-NAME group.
